# Knocking Out Antimicrobial Resistance editorial: reflections on the United Nations General Assembly high-level meeting on antimicrobial resistance

**DOI:** 10.1099/jmm.0.001937

**Published:** 2024-11-18

**Authors:** Catrin E. Moore, Lovleen Tina Joshi

**Affiliations:** 1 City St George’s, University of London, London, UK; 2 Peninsula Dental School, Faculty of Health, University of Plymouth, Plymouth, UK

**Keywords:** antimicrobial resistance, high-level meeting, Knocking Out AMR, Political Declaration, UNGA79, United Nations General Assembly

## Editorial

On 26 September 2024, the 79th United Nations General Assembly (UNGA) convened for the high-level meeting (HLM) on antimicrobial resistance (AMR) in New York, USA. Here, 193 world leaders approved a Political Declaration committing to specific actions and targets to reduce human deaths associated with AMR annually by 10% in 2030 [1]. This is the second Political Declaration on AMR action after the inaugural HLM on AMR in 2016 [2]. The declaration has formalized and recognized the leading roles of the Quadripartite Joint Secretariat on Antimicrobial resistance, comprising the World Health Organization (WHO), the Food and Agriculture Organization of the United Nations, the World Organization for Animal Health, the United Nations Environment Programme and the Global Leaders Group on AMR and AMR Multi-Stakeholder Partnership Platform.

The Microbiology Society attended the UNGA HLM meeting in person to witness the declaration as part of the AMR Multi-Stakeholder Partnership platform. We represented our 8000 global members and highlighted the international Knocking Out AMR project [3]. Knocking Out AMR puts microbiologists at the forefront of developing innovative solutions to the problem of AMR, as microbiologists are subject matter experts with the essential scientific background to offer scientific evidence and context to the AMR crisis across the One Health sphere.

The Political Declaration reviewed the progress on multi-sectorial global efforts to tackle AMR through a One Health approach to ensure that we live in a healthier, equitable world: thus, reaffirming and advancing the 2030 Agenda for the UN Sustainable Development Goals [4]. Key commitments included improving the appropriate and responsible use of antimicrobials across human, animal, and plant health via implementing disease prevention and antimicrobial stewardship policies; this related to implementing effective infection prevention and control measures for all the sectors and ensuring that 100% of countries have access to the clean water, sanitation, hygiene, and waste management (WASH). The declaration committed to invest in universal health facilities, infrastructure and stewardship programmes, improve vaccination and prevent infections through equitable access to medicine, diagnostics and support education from low- to middle-income countries (LMICs) and globally [1].

This is an important step in bringing a cohesive approach to the AMR policy globally, *if* the LMICs are represented, as AMR disproportionately affects the Global South. However, the UN HLM was slow to inform many participants from the Global South about their admission to the UNGA event, resulting in participants being unable to apply for visas in time [5]. This absence of timely communication resulted in a lack of equality, diversity and inclusion at the UN HLM, and the lack of representation of experts from the Global South was evident across the week’s events. The irony that this happened at the United Nations was not lost on those present.

The importance of preventing antimicrobial pollution was emphasized through reducing the discharge of antimicrobials into the environment. However, no measurable targets for the reduction of antimicrobial use in food or agriculture were identified in the declaration [1]. Measures to ensure animal vaccination and husbandry infection prevention were defined in the implementation plans, as was strengthening surveillance systems for AMR and antimicrobial use via the WHO Global Antimicrobial Resistance and Use Surveillance System (WHO GLASS) and other databases [6].

The current AMR research environment was also highlighted as being ‘inadequate and insufficient’ for the development of vaccines, diagnostics therapeutics, antimicrobials, and viable alternatives [1]. However, the global ‘brain drain’ of scientists and researchers from the field was overlooked [7]. We need innovation in AMR and also we need to encourage our early career researchers and industry scientists to stay in the field. This is why the Microbiology Society launched the Knocking Out AMR project to support the convening of global stakeholders to seek solutions to minimize AMR and to provide a platform to ensure that microbiologists from all backgrounds and career stages are heard.

Interestingly, a key financial target of the declaration is to facilitate sustainable funding and achieve a target of $100 million USD to ‘catalyse the achievement for 60% of countries’ to fund their National Action Plans by 2030 [1]. In the scheme of international corporate finance, government defence budgets and industry, $100 m seems like a small drop in the ocean. More investment and funding are needed to ensure action is taken to reduce the potential catastrophe of AMR across all sectors. For example, when we consider that, in comparison, the same amount of money (£100 million) was donated to create an AMR research institute at a leading university in the UK, the declaration of $100 million USD from 193 countries seems to fall short to deliver a global step change to minimize AMR [8]. The $100 million commitment was a disappointing outcome and a subject of debate at the HLM.

The declaration also detailed the formation of an independent panel of influential experts able to use globally shared data, delivering scientific evidence to advocate for the action on AMR, like the Intergovernmental Panel on Climate Change formed by the World Meteorological Organization and the United Nations Environment Programme in 1988 [9]. This news was welcome as an independent panel of influential AMR experts is long overdue. Perhaps, this will raise the profile of AMR and catalyse AMR action globally.

One clear message that came across all AMR events during UNGA79 was the importance of investing in the antimicrobial pipeline and in the development of new antimicrobials. Vaccines and diagnostics were also discussed but perhaps to a lesser extent by diplomats and key opinion leaders. This led us to question why there was so little focus on the microorganisms themselves. There was no mention of the role of microorganisms in the problem of AMR, nor was the biology, behaviour, transmission, and evolution of microorganisms mentioned. Alarmingly, a target for inappropriate antimicrobial use was excluded from the declaration and there appeared to be limited understanding of the long-term implications of inappropriate antimicrobial use and the amazing capacity microorganisms have to evolve to survive [1]. There was a disconcerting sense of optimism among diplomats that they were ‘going to solve the problem’ by working together globally. This was a noble notion, but we have been here before; therefore, this felt entirely unrealistic and somewhat naive, given the scale of the AMR crisis. Arguably, the horse has already bolted when it comes to AMR, and we cannot solve the microbial evolution.

The more antimicrobials we use, the more the microorganisms will find a way to survive. Microbiology is the heart of AMR, and many diplomats failed to grasp this. What was abundantly clear was that we, as microbiologists need to continue to speak and advocate for understanding the biology of AMR pathogens to effectively prevent, manage, and control AMR. We, as the experts, need to find a universal language so that our scientific knowledge and findings impact key leaders and diplomats and influence policy.

This is why the Microbiology Society’s Knocking Out AMR project is so important; we are championing both the discipline of microbiology and our expert members who understand the scientific drivers of AMR. We must continue to advocate and champion microbiology among the crowded voices in AMR; these voices are focusing on the host response only and are pushing microbiologists out of the AMR space. Microbes will continue to evolve, and microbiologists have an extremely important part to play in solving the AMR puzzle through scientific research, clinical research, and understanding pathogen behaviour.

The hope is that at the next UNGA HLM on AMR in New York in 2029, the microbiological themes underlying AMR are at the forefront of discussions and that microbiologists provide scientific evidence to inform the policy and support AMR advocacy. Indeed, including expert microbiologists on the new independent AMR panel would be a good start.

## Abbreviations

AMR: antimicrobial resistance
GLASS: global antimicrobial resistance and use surveillance system
HLM: high-level meeting
IPCC: intergovernmental panel on climate change
LMICs: low- to middle-income countries
UNGA: United Nations General Assembly
WASH: water, sanitation and hygiene
WHO: World Health Organization
